# Role of Nucleocapsid Protein Antigen Detection for Safe End of Isolation of SARS-CoV-2 Infected Patients with Long Persistence of Viral RNA in Respiratory Samples

**DOI:** 10.3390/jcm10184037

**Published:** 2021-09-07

**Authors:** Antonella Mencacci, Alessio Gili, Anna Gidari, Elisabetta Schiaroli, Carla Russo, Elio Cenci, Barbara Camilloni, Alessandro Graziani, Arduino Melelli-Roia, Daniela Francisci, Fabrizio Stracci

**Affiliations:** 1 Department of Medicine and Surgery, Microbiology and Clinical Microbiology, Santa Maria Della Misericordia” Hospital, University of Perugia, 06129 Perugia, Italy; carla.russo179@gmail.com (C.R.); elio.cenci@unipg.it (E.C.); barbara.camilloni@unipg.it (B.C.); grazianialessandro2@gmail.com (A.G.); arduino.melelliroia@studenti.unipg.it (A.M.-R.); 2 Public Health Section, Department of Medicine and Surgery, University of Perugia, 06129 Perugia, Italy; fabrizio.stacci@unipg.it; 3 Department of Medicine and Surgery, Infectious Diseases Clinic, “Santa Maria della Misericordia” Hospital, University of Perugia, 06129 Perugia, Italy; anna.gidari@studenti.unipg.it (A.G.); elisabetta.schiaroli@unipg.it (E.S.); daniela.francisci@unipg.it (D.F.)

**Keywords:** SARS-CoV-2, antigen test, RT-PCR, virus culture, isolation and discharge recommendations

## Abstract

Background. In SARS-CoV-2 infection, viral RNA may persist in respiratory samples for several weeks after the resolution of symptoms. Criteria to assess the end of infectivity are not unequivocally defined. In some countries, time from diagnosis is the unique criterion used, in addition to symptom cessation. This study evaluates the role of the Lumipulse^®^ Antigen Assay (LAA) for the safe end of isolation of patients ≥21 days after the diagnosis of infection. Methods. A total of 671 nasopharyngeal swabs from patients diagnosed with infection at least 21 days before were assessed by RT-PCR and LAA, and the role of LAA in predicting the absence of infectivity was evaluated by virus cell culture. Results. Viable virus was present in 10/138 cultured samples. Eight out of ten infective patients suffered from a concomitant disease, predisposing them to long-term shedding of infective virus. In particular, infectious virus was isolated from 10/20 RT-PCR+/LAA+ cultured samples, whereas no viable virus was found in all 118 RT-PCR+/LAA– cultured swabs. LLA and RT-PCR agreed in 484/671 (72.1%) samples, with 100% and 26.7% concordance in RT-PCR negative and positive samples, respectively. Conclusions. Viable virus can be found ≥21 days after diagnosis in immunocompromised or severely ill patients. LAA better than RT-PCR predicts non-infectivity of patients and can be safely used to end isolation in cases with long persistence of viral RNA in the respiratory tract.

## 1. Introduction

In SARS-CoV-2 infection, viral RNA may persist in respiratory samples for several weeks after the resolution of symptoms [1,2,3,4], but this does not necessarily indicate shedding of infective virions. The duration of infection and risk of viral transmission among different individuals is variable [5]. It is known that the decline in patient infectivity occurs rather quickly after SARS-CoV-2 infection, usually within one or two weeks since symptom onset [4,6,7]. A recent meta-analysis found that, although patients with SARS-CoV-2 infection might have prolonged RNA shedding of up to 83 days, no culturable virus was isolated beyond day 9 [8]. However, in immunocompromised or severely ill patients, detection of sub-genomic RNA or of replication-competent particles has been reported beyond 20 days, and as long as 143 days after a positive SARS-CoV-2 test result [9,10,11,12,13,14].

Health authorities have adopted different protocols to discontinue isolation of infected persons worldwide. Centers for Disease Control and Prevention guidelines rely on a symptomatic approach (i.e., in asymptomatic or mild-symptomatic patients, isolation and precautions can be discontinued 10 days after symptom onset, and in patients with severe illness, extended duration of isolation for up to 20 days after symptom onset is recommended) [15]. Similarly, according to European Centre for Disease Prevention and Control guidelines, asymptomatic people should self-isolate for 10 days from the date of the sample collection, and severely ill patients should be isolated for at least 14 and up to 20 days from the onset of symptoms, based on an individual case risk assessment [16]. Two consecutive negative SARS-CoV-2 RT-PCR tests, ideally in a 24-h period, are recommended for the discontinuation of isolation for immunocompromised or severely ill patients, especially in case of transfer to other hospital wards or discharge to a long-term care facility [16]. Current Italian guidelines are based on the SARS-CoV-2 variant causing the infection. For patients infected by a non-beta variant, a negative molecular or antigenic test is required to discontinue isolation 10 days after symptom onset, and a symptomatic approach is used after 21 days. In case of infections due to the beta variant, a negative molecular test is required to end isolation, regardless of the time elapsed [17].

Diagnostic methods to certify the non-infectious status of patients recovered from symptoms and discontinue isolation are not unanimously recognized. Indeed, the presence of culturable SARS-CoV-2 particles in respiratory samples can be considered a marker of infectivity, but virus culture is not widely applicable in routine diagnostics. RT-PCR can be used instead, but positive results can be obtained several weeks after the start of infection without viral growth in culture. To date, the correlation between RT-PCR results with viral cultures and contagiousness remains scarcely investigated [9]. The use of molecular detection of viral RNA implies the risk for many patients to remain hospitalized or isolated for a much longer time than necessary, with important implications for patient management, and also psychological, social, and economic consequences [18,19,20,21]. Therefore, availability of an accurate, rapid, and simple laboratory tool to certify the end of infectivity of SARS-CoV-2-infected patients would have a great impact.

Different antigenic tests have been released to diagnose SARS-CoV-2 infection, with different sensitivity. It has been reported that rapid antigen tests can detect individuals harboring infectious viruses [22]. Recent studies indicate that the Lumipulse^®^ quantitative antigen assay (LAA), given its optimal correlation with the RT-PCR test early in infection, can be safely employed in the screening strategies [23,24]. Interestingly, concordance between LAA and RT-PCR declines with lower viral loads and duration of infection [23,25,26]. Based on the observation that viral antigen declines more rapidly than RNA, we hypothesized that quantitative antigenic tests better than molecular tests could be of help in ruling out infectivity in the late phase of infection.

A previous study on 1738 cases evaluating LAA for screening of SARS-CoV-2 infection found 100% sensitivity and 51.1% positive predictive value (PPV) at the optimal antigen cutoff of 1.645 pg/mL [24]. The same study showed that the best positive likelihood ratio (LR+) was found at 10.4 pg/mL cutoff value, with 99.8% specificity [24]. Indeed, the manufacturer, according to the validation study of Hirotsu et al. [23], suggests that samples with an antigen concentration >10 pg/mL do not need to be confirmed for SARS-CoV-2 infection by a molecular test. Instead, samples with antigen load between 1.0 and 10 pg/mL need confirmation. Based on these data, we hypothesized that a cutoff of 10 pg/mL could be appropriate to exclude contagiousness.

The aim of the present study was to evaluate the presence of infectious patients among cases with documented infection diagnosed ≥21 days before and the need of testing to safely end isolation. Then, we assessed agreement between RT-PCR, the test commonly used to end patient isolation, and the LAA test. Both tests were compared to cell culture as the gold standard test of the presence of viable virus. Finally, we discussed the implications of our findings for the safe end of isolation in RNA long-term shedding patients.

## 2. Materials and Methods

### 2.1. Samples

Consecutive samples tested with both RT-PCR and LAA, collected from patients diagnosed with infection at least 21 days before, were included in the study. To include in the study both symptomatic and asymptomatic patients, days after infection were calculated starting from the first positive molecular test instead of the day of symptom onset. A total of 671 nasopharyngeal swabs, collected from 608 individuals with SARS-CoV-2 infection, analyzed from 1 October 2020 to 30 March 2021 at the Microbiology Unit of the Santa Maria della Misericordia Perugia General Hospital (the Umbria Regional Reference Laboratory for diagnosis of SARS-CoV-2 infection), were analyzed. For each patient, only the first RT-PCR-/LAA– sample was included, and subsequent double negative swabs were excluded (311 samples from 100 patients). Selected samples (138) were analyzed by cell culture, when knowledge of patient infectivity was important for patient management or monitoring (e.g., programmed hospital admission due to non-COVID-19-related diseases, hospital discharge of COVID-19 patients in long-term care facilities, re-admission of patients among non-infected family members, interruption of isolation, monitoring of treatment).

### 2.2. Testing

All nasopharyngeal swabs were tested for SARS-CoV-2 by RT-PCR and with a quantitative antigen test. Swabs were collected in Universal Transport Medium (UTM, Copan, Brescia, Italy). For RT-PCR, samples were analyzed by the Allplex™ SARS-CoV-2 assay (Seegene, Seoul, Korea), as previously described [24]. The envelope (E), the nucleocapsid (N), and the RNA-dependent-RNA-polymerase (RdRp) genes were the target genes. The amplification curves were visually assessed, and threshold cycle (Ct) values for each target gene were recorded. Ct value refers to the number of cycles required to amplify viral RNA to reach a detectable level. Thus, lower Ct values correspond to a higher viral load, while higher Ct values correspond to a lower viral load. For positive samples with different Ct values for the three target genes, the lowest Ct was considered for the analysis. For the antigen test, after removal of the swabs, UTM tubes were centrifuged at 1400× *g* for 10 min and loaded on the Lumipulse^®^ G1200 automated immunoassay analyzer (Fujirebio) to measure the nucleocapsid protein (NP) antigen level (pg/mL) with the Lumipulse^®^ SARS-CoV-2 antigen kit (Fujirebio), as previously described [24]. A cutoff of 10 pg/mL was considered for LLA test positivity.

### 2.3. Viral Culture

Viral growth in cell culture was assessed in the biosafety level-3 facility of the virology laboratory of the Infectious Diseases Clinic of the Santa Maria della Misericordia Perugia General Hospital, as described elsewhere [27]. Samples tested positive for the SARS-CoV-2 on RT-PCR were mixed with nystatin (10,000 U/mL, 1:1 ratio) and penicillin–streptomycin (10,000 U/mL, 1:4 ratio) and left to react at 4 °C for 1 h. Samples were centrifuged at 400× *g* for 10 min, and the supernatant was used to inoculate Vero E6 (ATCC^®^-1586) cells, maintained at 37 °C in the presence of 5% CO_2_ in Eagle’s essential medium, supplemented with 10% fetal bovine serum and 1% penicillin–streptomycin. Cells were cultured for 5 days, as described above. Viral replication was evaluated through the cytopathic effect, observed at different exposure times, and RT-PCR tests on cell culture supernatant [28,29].

### 2.4. Statistical Analysis

LAA was compared in terms of agreement with RT-PCR. Viral growth in cell culture was considered the gold standard to assess contagious infection. Samples were considered positive for SARS-CoV-2 RNA if at least one of the target genes was detected (any cycle threshold, Ct). For the antigen assay, the cutoff value of 10 pg/mL was considered to discriminate swabs positive for SARS-CoV-2 antigen from negative samples, based on previous studies [23,24,26] and according to the manufacturer’s instructions. Descriptive statistics were calculated, including percentages, mean ± standard deviation (SD), median, interquartile range (IQR), and range. A non-parametric Mann–Whitney test was performed to compare continuous variables with non-normal distribution. Test sensitivity, specificity, ROC area, Positive Likelihood Ratio (LR+) and Negative Likelihood Ratio (LR–), Positive Predicted Value (PPV), and Negative Predicted Value (NPV) were calculated to compare the LAA test with cell culture or RT-PCR. The relationship between RT-PCR Ct levels and LAA antigen concentration was assessed by linear regression. We also evaluated a locally weighted regression [30] and restricted cubic spline [31]. The likelihood ratio test (LRT) was used to evaluate the goodness-of-fit between three models. A *p*-value < 0.05 was considered statistically significant. Results were analyzed using Stata Statistical Software (Release 16.1, College Station, Houston, TX, USA: StataCorp LLC).

## 3. Results

During the study period, 671 nasopharyngeal swabs from 608 patients, collected ≥21 days after the molecular diagnosis of SARS-CoV-2 infection, were tested by both RT-PCR and LAA. RT-PCR and LLA gave concordant results for 484/671 (72.1%) samples. Among 255 samples testing positive on RT-PCR, only 68 were positive for the antigen (26.7% concordance). All samples negative on RT-PCR were also negative on the antigen test, with 100% concordance (Table 1).

**Figure 1:**
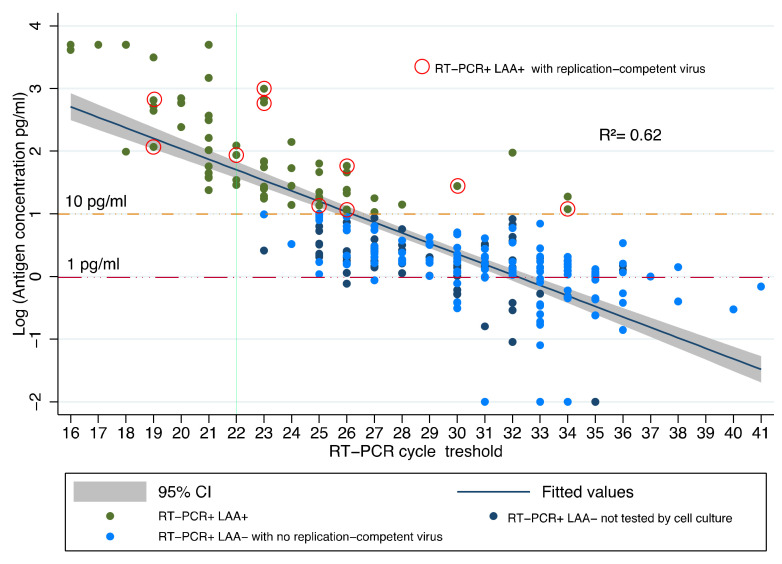
Relationship between Ct values and NP antigen concentration (log_10_, pg/mL) in 255 samples that, 21 days or more after diagnosis, were still positive on RT-PCR. See Table 3 below for details on RT-PCR+/LAA+ samples negative for replication-competent virus.

The time interval between the date of molecular diagnosis and study samples was significantly longer (*p* < 0.01, Mann–Whitney) in the RT-PCR–/LAA– than RT-PCR+/any LAA samples. Figure 1 shows the relationships between Ct values and antigen concentration in 255/671 RNA positive samples (*R*^2^ = 0.62). Linear regression coefficient beta was −3.7 (95% CI from −4.1 to −3.2). LRT was not statistically significant comparing locally weighted regression and restricted cubic spline with the linear approach (LRT 0.378 and 0.455, respectively).

While all samples with Ct ≤ 22 were positive for the antigen (i.e., antigen concentration ≥ 10 pg/mL), samples with Ct > 22 corresponded to both positive or negative LAA. Samples ≥35 Ct were all LAA negative.

The mean Ct value for RT-PCR+/LAA+ samples (*n* = 68) was 23.0 ± 3.7 SD (median 23.0, IQR 4), a value significantly lower (*p* < 0.01, Mann–Whitney) than that observed among 187 RT-PCR+/LAA– samples (mean 30.5 ± 3.55 SD; median 31.0, IQR 6). The mean value of antigen concentration in RT-PCR+/LAA+ samples was 627.0 pg/mL ± 1,402.0 SD (median 56.4 pg/mL, IQR 383.0), and in RT-PCR+/LAA– samples was 2.2 pg/mL ± 2.2 SD (median 1.6, IQR 1.62).

A total of 138/255 (54.1%) RT-PCR positive samples were tested for virus viability by cell culture, including 20/68 (29.4%) RT-PCR+/LAA+ concordant samples and 118/187 (63.1%) RT-PCR+/LAA– discordant samples. Viable SARS-CoV-2 virus was isolated from 10/20 (50%) RT-PCR+/LAA+ samples, and no replication-competent virus (0%) was found among 118/118 RT-PCR+/LAA– swabs (Table 2).

Results from 20 RT-PCR+/LAA+ cultured samples, collected from 9 hospitalized patients and 11 outpatients, are detailed in Table 3.

Eight of 10 positive and 1 of 10 negative cultured samples were from hospitalized patients. Infectious samples were from critically ill patients with severe COVID-19 infection (two cases), or from patients with concomitant onco-hematologic diseases (three cases), diabetes (one case), dialysis (one case), solid cancer (one case), hepatitis (one case), and from one patient under corticosteroid treatment. Time from diagnosis in RT-PCR+/LAA+ samples with viable virus (median 24.5 days, IQR 20) was not significantly different (*p* = 0.18) from that of RT-PCR+/LAA+ samples without viable virus (median 37 days, IQR 12). Median antigen concentration of the culture positive group (79.2 pg/mL, IQR 573.2) was not significantly different (*p* = 0.08) from that of the culture negative one (17.1 pg/mL, IQR 21.1). Similarly, median Ct values in the two groups were not significantly different (not shown).

A great difference of sensitivity of LAA in predicting the RT-PCR or culture results has been found, with 100% NPV of LAA in predicting negative viral culture (Table 4).

**Figure 2:**
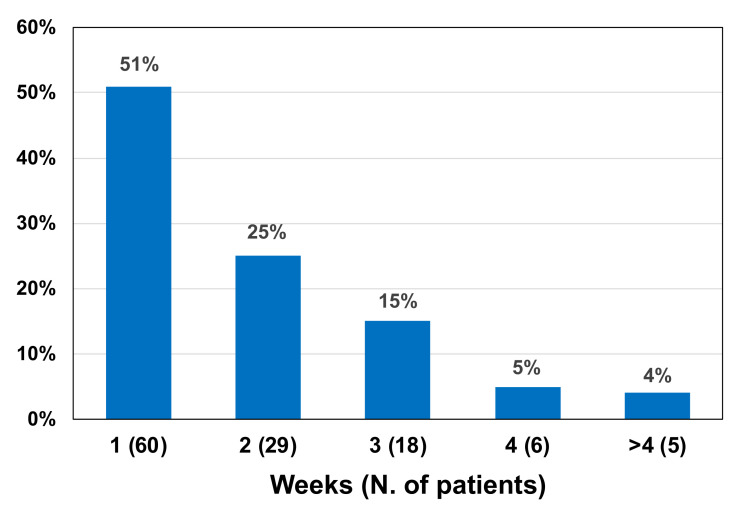
Time to negativity of the RT-PCR test in 118 patients with RT-PCR+/LAA– samples and negative for infectivity on cell culture. Time to negativity is the interval time between the first RT-PCR+/LAA– and the first RT-PCR-/LAA– sample.

One hundred eighteen patients with RT-PCR+/LAA– samples, negative for infectivity on cell culture, were further evaluated weekly with the molecular test until the first negative RT-PCR result was obtained. It was found that the molecular test switched from positive to negative within 2 weeks in 89/118 (74.6%) patients, and in an even longer time in the remaining cases (Figure 2).

## 4. Discussion

We found that the shedding of the viable virus occurs even after 21 days from diagnosis. This finding is in contrast with previous data reviewed by Cevik et al. [8]. Many studies indicate that positive RT-PCR samples collected several weeks after symptom onset or after recovery can reflect the presence of non-infectious viral debris and not viable and infectious SARS-CoV-2 [6,7,32,33,34,35]. Manzulli et al. demonstrated that patients showed the viral clearance at viral culture three days or more after clinical recovery [36]. However, other studies reported the persistence of active infection and contagiousness in immunocompromised or severely ill patients [9,11,12,13,14,37,38,39]. In line with these studies, long term shedding of viable virus was infrequent in our study (10 out of 138 cultured samples, 7.2%) and was nearly limited to patients with conditions associated with prolonged RT-PCR positivity and/or severe COVID-19, but the risk of transmission was still present after three weeks from diagnosis. Thus, patient testing to exclude infection persistence and risk of transmission should not be dismissed, particularly in cases of immunocompromised patients and hospitalized or community-dwelling elderly, but also for monitoring the response to treatment, and hospital discharge or admission.

Live virus isolation on cell culture is considered a reliable method to verify infectivity of patients with high NPV and remains the reference method. However, it is not easily applicable in the laboratory and clinical practice, due to the need for special laboratory equipment and trained personnel, and long time-to-results (≥5 days). Different guidelines indicate RT-PCR as the test of choice, not only for the diagnosis, but also for monitoring SARS-CoV-2 infection [15,16]. However, as previously mentioned, a problem with RT-PCR-based testing is the risk of long-term positivity in recovered patients without viable virus. Prolonged isolation of these patients has a great importance in terms of social, phycological, and economic consequences [18,19,20,21]. Moreover, these patients, despite being non-contagious, cannot access COVID-free healthcare facilities, and are at risk of re-infection, especially when new virus variants emerge, if admitted to COVID hospital wards.

Thus, as previously highlighted [40], there is a pressing need for a fast, easy to use in the laboratory work-flow, and an economically advantageous test to rule out infectivity in viral RNA long-shedding patients. This study suggests that LAA could be a suitable test for this purpose and could overcome RT-PCR in clinical practice. We found that 21 days after diagnosis, many samples (255/671, 38%) were still positive on the molecular test, but the majority of these (187/255, 73.3%) were negative on the antigen test, a finding which is in line with previous studies [26].

All cultured 118 RT-PCR+/LAA– samples resulted negative for viable virus, and 10 samples that grew in culture gave positive results both in RT-PCR and LAA. Bullard et al., in addition to excluding infectivity 8 days after symptom onset, showed that SARS-CoV-2 cell infectivity was only observed for samples with Ct <24 [7]. Arons et al., comparing RT-PCR results to cell culture in pre-symptomatic patients, reported no correlation between Ct values and viral growth, observed also in samples with >30 Ct [41]. We found infectious samples also with Ct >24, but no sample with <10 pg/mL antigen concentration carried viable virus (Figure 1). Thereby, LAA, compared with RT-PCR, greatly reduced the risk of false positivity without losing true positive cases.

Antigen declined faster than RNA in nasopharyngeal swabs. Based on the result of the antigen level <10 pg/mL, a gain of 2 weeks would have been obtained to end the patient’s isolation for about 75%, and even more than 2 weeks for the remaining 25% of cases (Figure 2). This implies that unnecessary isolation can be avoided using LAA testing with relevant advantages for patients, the health service, and society as a whole. Indeed, given a PPV of 50%, even LAA can overestimate the infectivity of patients. Nevertheless, the antigen test remains cost-effective compared to RT-PCR, being less expensive, faster (processing up to 120 samples per hour, with a turnaround time of about 1h), and automated [24].

The cut off value of the antigen assay considered in this study was 10 pg/mL. In the early phase of the infection, a ten-fold lower cut-off can be safely used for the screening strategies of SARS-CoV-2 infection [23,24,25]. Thus, antigen concentration between 1.0 and 10 pg/mL can predict a contagious acute status in the early phase of infection or recovery and non-infectivity in the late phase, while a concentration <10 pg/mL can be used to exclude infectivity only in patients diagnosed with infection at least 21 days before. Further studies are needed to assess the optimal testing strategy in the time period from 9 to 21 days after diagnosis, which was not considered in our study, and to explore the possible role of other quantitative antigenic assays.

A major limitation of the study was that samples were not population-based but reflected the activity of the main regional laboratory. Another limitation was the lack of information on clinical data and patient conditions related to RT-PCR and LAA results, except for 10 cases with viable virus. Third, in spite of a high number of RT-PCR+/LAA– samples evaluated for virus replication, only 20 RT-PCR+/LAA+ samples were cultured, and 10/20 harbored infectious virus. Indeed, even a limited number of samples was sufficient to confirm that 21 days or more after diagnosis, only samples with antigen concentration <10 pg/mL can be safely considered non-contagious.

## 5. Conclusions

Quantitative Lumipulse^®^ antigen assay, performed at least 21 days after the molecular diagnosis of SARS-CoV-2 infection, can be used to avoid unnecessary isolation in a number of RT-PCR positive, non-infectious, cases and thus can be the test of choice to assess the end of infectivity in SARS-CoV-2 patients.

## Figures and Tables

**Table 1:** Comparison of Lumipulse^®^ Antigen Assay (LAA) and RT-PCR results in 671 nasopharyngeal swabs, collected ≥21 days after the molecular diagnosis of SARS-CoV-2 infection. The cutoff of 10 pg/mL was considered to discriminate LAA positive and negative samples. Data are reported as numbers and percentages.

	LAA Positive	LAA Negative	Total
RT-PCR positive	68 (26.7%)	187 (73.3%)	255 (100.0%)
RT-PCR negative	0 (0%)	416 (100.0%)	416 (100.0%)
Total	68 (10.1%)	603 (89.9%)	671 (100%)

**Table 2:** Values of cycle threshold (Ct) and NP antigen concentration (pg/mL) in 138 samples assessed for infectivity by cell culture, and in samples resulted positive for viable virus.

	Samples Assessed by Culture			Samples with Viable Virus		
Sample Group	Number	Ct Mean (Range)	Antigen Mean (Range)	Number (%)	Ct Mean (Range)	Antigen Mean (Range)
RT-PCR+/LAA+	20	24.9 (19–34)	146.6 * (13.6–990.1)	10 (50%)	24.7 (19–33)	263.1 (13.6–990.1)
RT-PCR+/LAA–	118	31 (22–41)	1.38 (0.4–9.6)	0 (0%)	NA	NA

* *p* < 0.01 (RT-PCR+/LAA+ vs. RT-PCR+/LAA– samples, Mann–Whitney test); NA, not applicable.

**Table 3:** Culture results from 20 RT-PCR+/LAA+ samples, collected 21 days or more after the first molecular diagnosis of SARS-CoV-2 infection.

Culture Result	Patient Age (Years)	Hospitalization	Days after Diagnosis	Ct Value	Antigen Level (pg/mL)
Positive	73	Yes	22	30	17.5
	33	No	23	26	19.8
	51	Yes	26	34	14.7
	45	Yes	22	22	93.2
	48	Yes	25	26	65.2
	76	Yes	23	19	732.2
	88	Yes	60	25	13.6
	47	No	24	23	990.1
	86	Yes	43	19	94.3
	46	Yes	48	23	590.7
Negative	72	No	39	28	15.5
	65	No	47	25	13.9
	73	No	37	34	18.8
	75	No	38	28	14.2
	76	Yes	25	23	14.4
	46	No	25	23	90.0
	81	No	27	22	31.2
	86	No	40	20	35.5
	90	No	35	22	52.3
	88	No	37	25	14.6

**Table 4:** Evaluation of Lumipulse^®^ antigen assay with 10.0 pg/mL cutoff to predict RT-PCR or culture results, 21 days or more after the first positive RT-PCR sample.

	vs. RT-PCR (95% C.I.)	vs. Culture (95% C.I.)
Sensitivity	26.7% (21.3–32.5)	100% (69.0–100)
Specificity	100.0% (99.1–100)	92.2% (86.1–96.2)
NPV	69.0% (65.1–72.7)	100% (96.1–100)
PPV	100.0% (94.7–100)	50.0% (35.6–64.5)
LR+	NA	12.8 (7.1–23.2)
LR–	0.73 (0.68–0.79)	NA
AUC	63.2% (61.4–66.3)	92.7% (87.1–96.5)

Notes: AUC, Area Under Curve; LR+, Positive Likelihood Ratio; LR–, Negative Likelihood Ratio; NA, Not Applicable; NPV, Negative Predictive Value; PPV, Positive Predictive Value.

## Data Availability

The data presented in this study are available upon request from the corresponding authors.

## References

[1] Peng J., Wang M., Zhang G., Lu E. (2020). Seven discharged patients turning positive again for SARS-CoV-2 on quantitative RT-PCR. Am. J. Infect. Control..

[2] Fu W., Chen Q., Wang T. (2020). Letter to the Editor: Three cases of re-detectable positive SARS-CoV-2 RNA in recovered COVID-19 patients with antibodies. J. Med. Virol..

[3] Woodruff A. (2020). COVID-19 follow up testing. J. Infect..

[4] Liu W.D., Chang S.Y., Wang J.T., Tsai M.J., Hung C.C., Hsu C.L., Chang S.C. (2020). Prolonged virus shedding even after seroconversion in a patient with COVID-19. J. Infect..

[5] Cento V., Colagrossi L., Nava A., Lamberti A., Senatore S., Travi G., Rossotti R., Vecchi M., Casati O., Matarazzo E. (2020). Persistent positivity and fluctuations of SARS-CoV-2 RNA in clinically-recovered COVID-19 patients. J. Infect..

[6] Wölfel R., Corman V.M., Guggemos W., Seilmaier M., Zange S., Müller M.A., Niemeyer D., Jones T.C., Vollmar P., Rothe C. (2020). Virological assessment of hospitalized patients with COVID-2019. Nature.

[7] Bullard J., Dust K., Funk D., Strong J.E., Alexander D., Garnett L., Boodman C., Bello A., Hedley A., Schiffman Z. (2020). Predicting Infectious Severe Acute Respiratory Syndrome Coronavirus 2 From Diagnostic Samples. Clin. Infect. Dis..

[8] Cevik M., Tate M., Lloyd O., Maraolo A.E., Schafers J., Ho A. (2021). SARS-CoV-2, SARS-CoV, and MERS-CoV viral load dynamics, duration of viral shedding, and infectiousness: A systematic review and meta-analysis. Lancet Microbe.

[9] Badu K., Oyebola K., Zahouli J.Z.B., Fagbamigbe A.F., de Souza D.K., Dukhi N., Amankwaa E.F., Tolba M.F., Sylverken A.A., Mosi L. (2021). SARS-CoV-2 Viral Shedding and Transmission Dynamics: Implications of WHO COVID-19 Discharge Guidelines. Front. Med..

[10] Aydillo T., Gonzalez-Reiche A.S., Aslam S., van de Guchte A., Khan Z., Obla A., Dutta J., van Bakel H., Aberg J., García-Sastre A. (2020). Shedding of Viable SARS-CoV-2 after Immunosuppressive Therapy for Cancer. N. Engl. J. Med..

[11] Avanzato V.A., Matson M.J., Seifert S.N., Pryce R., Williamson B.N., Anzick S.L., Barbian K., Judson S.D., Fischer E.R., Martens C. (2020). Case study: Prolonged infectious SARS-CoV-2 shedding from an asymptomatic immunocompromised individual with cancer. Cell.

[12] Baang J.H., Smith C., Mirabelli C., Valesano A.L., Manthei D.M., Bachman M.A., Wobus C.E., Adams M., Washer L., Martin E.T. (2021). Prolonged Severe Acute Respiratory Syndrome Coronavirus 2 Replication in an Immunocompromised Patient. J. Infect. Dis..

[13] Choi B., Choudhary M.C., Regan J., Sparks J.A., Padera R.F., Qiu X., Solomon I.H., Kuo H.H., Boucau J., Bowman K. (2020). Persistence and Evolution of SARS-CoV-2 in an Immunocompromised Host. N. Engl. J. Med..

[14] Tarhini H., Recoing A., Bridier-Nahmias A., Rahi M., Lambert C., Martres P., Lucet J.C., Rioux C., Bouzid D., Lebourgeois S. (2021). Long term SARS-CoV-2 infectiousness among three immunocompromised patients: From prolonged viral shedding to SARS-CoV-2 superinfection. J. Infect. Dis..

[15] Centers for Disease Control and Prevention Interim Guidance on Ending Isolation and Precautions for Adults with COVID-19. https://www.cdc.gov/coronavirus/2019-ncov/hcp/duration-isolation.html.

[16] European Center for Disease Prevention and Control Guidance for Discharge and Ending of Isolation of People with COVID-19. https://www.ecdc.europa.eu/en/publications-data/covid-19-guidance-discharge-and-ending-isolation.

[17] Circolare del Ministero Della Salute Aggiornamento Sulle Misure di Quarantena e di Isolamento Raccomandate Alla Luce Della Circolazione Delle Nuove Varianti SARS-CoV-2 in Italia ed in Particolare Della Diffusione Della Variante Delta (Lignaggio B.1.617.2). https://fimmg.bari.it/documenti/FDRBC_1.pdf.

[18] Rodríguez-Fernández P., González-Santos J., Santamaría-Peláez M., Soto-Cámara R., Sánchez-González E., González-Bernal J.J. (2021). Psychological Effects of Home Confinement and Social Distancing Derived from COVID-19 in the General Population-A Systematic Review. Int. J. Environ. Res. Public. Health.

[19] Bashir M.F., Ma B., Shahzad L. (2020). A brief review of socio-economic and environmental impact of Covid-19. Air Qual. Atmos. Health.

[20] Nicola M., Alsafi Z., Sohrabi C., Kerwan A., Al-Jabir A., Iosifidis C., Agha M., Agha R. (2020). The socio-economic implications of the coronavirus pandemic (COVID-19): A review. Int. J. Surg..

[21] Brodeur A., Gray D., Islam A., Bhuiyan S. (2021). A literature review of the economics of COVID-19. J. Econ. Surv..

[22] Kohmer N., Rabenau H.F., Hoehl S., Kortenbusch M., Ciesek S., Berger A. (2021). Comparative analysis of point-of-care, high-throughput and laboratory-developed SARS-CoV-2 nucleic acid amplification tests (NATs). J. Virol. Methods.

[23] Hirotsu Y., Maejima M., Shibusawa M., Nagakubo Y., Hosaka K., Amemiya K., Sueki H., Hayakawa M., Mochizuki H., Tsutsui T. (2020). Comparison of automated SARS-CoV-2 antigen test for COVID-19 infection with quantitative RT-PCR using 313 nasopharyngeal swabs, including from seven serially followed patients. Int. J. Infect. Dis..

[24] Gili A., Paggi R., Russo C., Cenci E., Pietrella D., Graziani A., Stracci F., Mencacci A. (2021). Evaluation of Lumipulse^®^ G SARS-CoV-2 antigen assay automated test for detecting SARS-CoV-2 nucleocapsid protein (NP) in nasopharyngeal swabs for community and population screening. Int. J. Infect. Dis..

[25] Hirotsu Y., Maejima M., Shibusawa M., Amemiya K., Nagakubo Y., Hosaka K., Sueki H., Hayakawa M., Mochizuki H., Tsutsui T. (2021). Analysis of a persistent viral shedding patient infected with SARS-CoV-2 by RT-qPCR, FilmArray Respiratory Panel v2.1, and antigen detection. J. Infect. Chemother..

[26] Hirotsu Y., Maejima M., Shibusawa M., Amemiya K., Nagakubo Y., Hosaka K., Sueki H., Hayakawa M., Mochizuki H., Tsutsui T. (2021). Prospective study of 1308 nasopharyngeal swabs from 1033 patients using the LUMIPULSE SARS-CoV-2 antigen test: Comparison with RT-qPCR. Int. J. Infect. Dis..

[27] Gidari A., Nofri M., Saccarelli L., Bastianelli S., Sabbatini S., Bozza S., Camilloni B., Fusco-Moffa I., Monari C., De Robertis E. (2021). Is recurrence possible in coronavirus disease 2019 (COVID-19)? Case series and systematic review of literature. Eur. J. Clin. Microbiol. Infect. Dis..

[28] Keyaerts E., Vijgen L., Maes P., Neyts J., Van Ranst M. (2005). Growth kinetics of SARS-coronavirus in Vero E6 cells. Biochem. Biophys. Res. Commun..

[29] Kim J.M., Kim H.M., Lee E.J., Jo H.J., Yoon Y., Lee N.J., Son J., Lee Y.J., Kim M.S., Lee Y.P. (2020). Detection and Isolation of SARS-CoV-2 in Serum, Urine, and Stool Specimens of COVID-19 Patients from the Republic of Korea. Osong Public Health Res. Perspect..

[30] Cleveland W.S. (1979). Robust locally weighted regression and smoothing scatterplots. J. Am. Stat. Assoc..

[31] Harrell F.E. (2001). Regression Modeling Strategies: With Applications to Linear Models, Logistic Regression, and Survival Analysis.

[32] La Scola B., Le Bideau M., Andreani J., Hoang V.T., Grimaldier C., Colson P., Gautret P., Raoult D. (2020). Viral RNA load as determined by cell culture as a management tool for discharge of SARS-CoV-2 patients from infectious disease wards. Eur. J. Clin. Microbiol. Infect. Dis..

[33] Million M., Lagier J.C., Gautret P., Colson P., Fournier P.E., Amrane S., Hocquart M., Mailhe M., Esteves-Vieira V., Doudier B. (2020). Early treatment of COVID-19 patients with hydroxychloroquine and azithromycin: A retrospective analysis of 1061 cases in Marseille, France. Travel Med. Infect. Dis..

[34] Perera R.A.P.M., Tso E., Tsang O.T.Y., Tsang D.N.C., Fung K., Leung Y.W.Y., Chin A.W.H., Chu D.K.W., Cheng S.M.S., Poon L.L.M. (2020). SARS-CoV-2 virus culture and subgenomic RNA for respiratory specimens from patients with mild coronavirus disease. Emerg. Infect. Dis..

[35] van Kampen J.J.A., van de Vijver D.A.M.C., Fraaij P.L.A., Haagmans B.L., Lamers M.M., Okba N., van den Akker J.P.C., Endeman H., Gommers D.A.M.P.J., Cornelissen J.J. (2021). Duration and key determinants of infectious virus shedding in hospitalized patients with coronavirus disease-2019 (COVID-19). Nat. Commun..

[36] Manzulli V., Scioscia G., Giganti G., Capobianchi M.R., Lacedonia D., Pace L., Cipolletta D., Tondo P., De Nittis R., Rondinone V. (2021). Real Time PCR and Culture-Based Virus Isolation Test in Clinically Recovered Patients: Is the Subject Still Infectious for SARS-CoV2?. J. Clin. Med..

[37] Zhou Y., Ding F., Bao W., Xue Y., Han L., Zhang X., Zhang P., Ji Y., Yin D., Bao A. (2021). Clinical features in coronavirus disease 2019 (COVID-19) patients with early clearance and prolonged shedding of severe acute respiratory syndrome coronavirus 2 (SARS-CoV-2) RNA. Ann. Transl. Med..

[38] Kim M.C., Cui C., Shin K.R., Bae J.Y., Kweon O.J., Lee M.K., Choi S.H., Jung S.Y., Park M.S., Chung J.W. (2021). Duration of Culturable SARS-CoV-2 in Hospitalized Patients with Covid-19. N. Engl. J. Med..

[39] Helleberg M., Niemann C.U., Moestrup K.S., Kirk O., Lebech A.M., Lane C., Lundgren J. (2020). Persistent COVID-19 in an immunocompromised patient temporarily responsive to two courses of remdesivir therapy. J. Infect. Dis..

[40] Manabe Y.C., Sharfstein J.S., Armstrong K. (2020). The Need for More and Better Testing for COVID-19. JAMA.

[41] Arons M.M., Hatfield K.M., Reddy S.C., Kimball A., James A., Jacobs J.R., Taylor J., Spicer K., Bardossy A.C., Oakley L.P. (2020). Public Health–Seattle and King County and CDC COVID-19 Investigation Team. Presymptomatic SARS-CoV-2 Infections and Transmission in a Skilled Nursing Facility. N. Engl. J. Med..

